# Tracheobronchopathia Osteochondroplastica: A Rare, Underrecognized Entity

**DOI:** 10.7759/cureus.28832

**Published:** 2022-09-06

**Authors:** Paras M Patel, Mallory E Jean, Kenzie Reich, Ojas Kaveeshwar, Suhani P Patel

**Affiliations:** 1 Interventional Pulmonology, John Peter Smith Hospital / TCU and UNTHSC School of Medicine, Fort Worth, USA; 2 Medical School, School of Health Sciences, John Hopkins University, Baltimore, USA; 3 Interventional Pulmonology, John Peter Smith Hospital, Fort Worth, USA; 4 Health Sciences, Reedy School, Frisco, USA; 5 Health Sciences, Carroll Middle School, Southlake, USA

**Keywords:** osseous protrusions, flattening of flow volume loop, nd:yag laser, interventional pulmonologist, tracheal stenosis, calcification of trachea, sparing posterior wall of trachea, tracheobronchopathia osteochondroplastica

## Abstract

Tracheobronchopathia Osteochondroplastica (TO) is an extremely rare condition characterized by the presence of nodules made of bone and cartilage within the submucosa of the tracheobronchial wall. These protuberant nodules inside the trachea and bronchi can lead to airway obstruction, resulting in patients who experience recurrent respiratory systems and infections. The exact etiology is unknown. The mean age of diagnosis is in the 5th - 6th decades of life. TO is often confused with other diagnoses, especially asthma. We report a 41-year-old female who presented with intermittent exertional dyspnea for 10 years. Workups, including pulmonary function test, CT chest, and most importantly, flexible bronchoscopy, aided in the appropriate diagnosis. The unique feature observed during bronchoscopy is the sparing of the posterior wall of the trachea and bronchi.

## Introduction

Tracheobronchopathia Osteochondroplastica (TO) is a non-malignant condition affecting the lumen of the tracheobronchial tree. It is characterized by abnormal chondrification and ossification of cartilages on the anterior and lateral walls, sparing the posterior wall [1]. TO is also referred to as tracheopathia chondroosteoplastica and tracheopathia osteoplastica [2].

TO was first described in 1857 by Wilks and colleagues as ossific deposits in the larynx, trachea, and bronchi [3]. Narrowing of the airways is caused by the accumulation of calcium phosphate in the submucosa of the large airways as well as the benign proliferation of bone and cartilage. TO is likely more frequent than it has been reported, but frequently underrecognized by most clinicians as it can be asymptomatic or present with nonspecific respiratory symptoms such as chronic cough and wheezing. These presenting symptoms of TO result in frequent misdiagnosis of asthma.

## Case presentation

**Figure 1:**
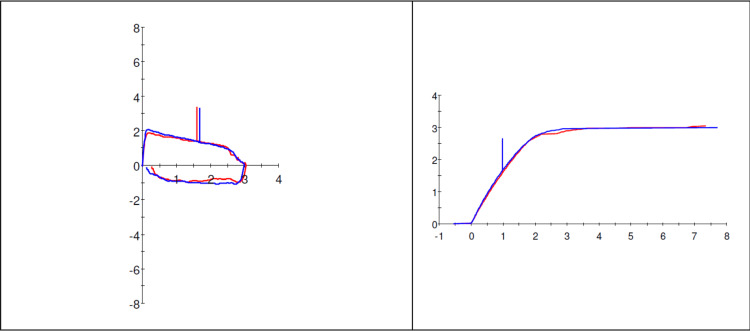
Flow volume loop Flow volume loop demonstrates the flattening of both inspiratory and expiratory limbs.

A 41-year-old female with a past medical history of obesity (BMI 49.6 kg/m2), gastroesophageal reflux disease, and asthma was referred to our Pulmonary clinic for evaluation of worsening exertional dyspnea for the last 10 years. She was appropriately treated for her asthma but continued to experience worsening dyspnea. She reported a 20-pound weight loss with diet/exercise but no improvement in her symptoms. A review of systems was negative for cough, hemoptysis, or chest pain. She had no seasonal allergies or rhinitis symptoms. A full pulmonary function test (PFT) was performed. Flow volume loops demonstrated flattening of both inspiratory and expiratory limbs suggestive of fixed upper airway obstruction (Figure 1).

**Figure 2:**
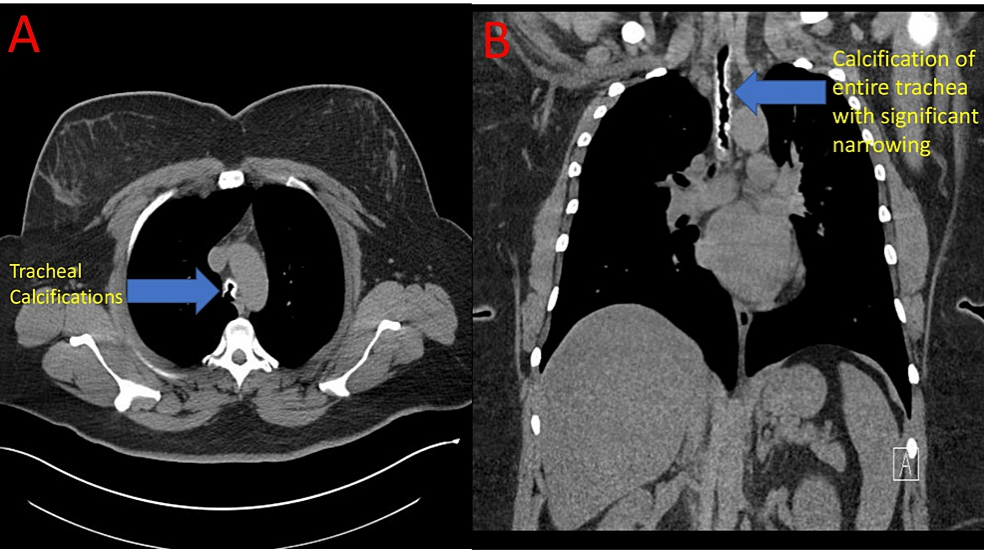
CT chest (A - axial view, B - coronal view) Inspiratory images from the CT scan of the chest ( A, B). Image A demonstrates tracheal calcifications (blue arrow). Image B demonstrates calcifications of the entire trachea with resultant severe tracheal narrowing (blue arrow).

Computerized tomography (CT) of the neck/chest was performed which revealed severe tracheal lumen narrowing with prominent/extensive calcifications along the tracheal wall. The trachea measured 4 to 5 mm in diameter in the narrowest portion (Figure 2).

**Figure 3:**
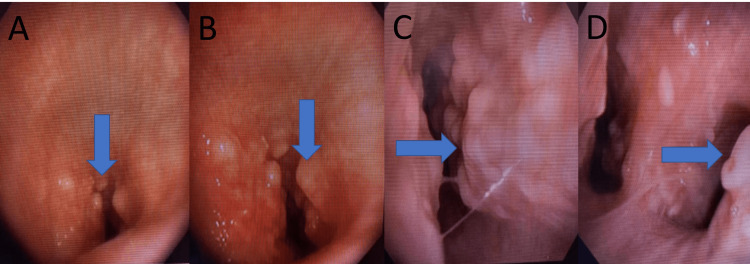
Bronchoscopic images Bronchoscopic images above (A, B, C, D) demonstrate osseous protrusions (blue arrow) covering the entire trachea and extending into the right and left mainstream bronchus. Images A and B represent the subglottic space and proximal trachea. Image C represents the mid trachea. Image D represents the main carina with a view into the right and left mainstream bronchus.

Flexible bronchoscopy was performed which revealed significant narrowing of the tracheal lumen from these osseous protrusions throughout the length of the trachea starting from the subglottic space and extending up to right and left mainstem bronchus. These nodular lesions were very firm/hard and difficult to biopsy. Histopathologic results included the presence of chronic inflammation, ossification, and cartilage formation. The key finding in bronchoscopy was the sparing of the posterior wall of the trachea (Figure 3).

A video of the bronchoscopy procedure was obtained. Video of the entire trachea shows significant narrowing from these osseous nodules making it difficult for the regular bronchoscope (2.0 mm working channel) to pass through the trachea. These nodules are seen covering the entire trachea up to right and left mainstream bronchus. The posterior wall of the trachea was spared and normal (Video 1).

**Video 1:** Bronchoscopic video demonstrating nodular lesions covering the entire trachea with sparing of the posterior wall

## Discussion

Tracheobronchopathia Osteochondroplastica (TO) is an extremely rare, benign condition affecting the central tracheobronchial tree but does not affect the lung parenchyma or other organs. TO is often under-diagnosed or diagnosis is delayed as most patients are asymptomatic or mistaken for other pulmonary conditions due to its association with non-specific respiratory symptoms such as chronic cough, wheezing/stridor, dyspnea, chest pain, hoarseness and hemoptysis [4]. An incidence of 0.01 to 4.2 per 100,000 has been estimated, with no difference in gender distribution [5]. The exact pathology of this condition is unknown but there have been two postulated hypotheses about the pathogenesis. The first one proposed by Virchow is that TO is caused by an exostosis [6]. The second one proposed by Aschoff is that TO is caused by ossification of the tracheal elastic connective tissue [7].

The diagnosis is primarily made by radiographic studies which include a CT scan of the neck and chest and direct visualization of the tracheobronchial tree via flexible bronchoscopy. Bronchoscopy is the gold standard for diagnosis, as it allows for direct visualization and estimation of the disease severity by providing means of observing airway obstruction and biopsies which are sent for histopathological analysis for diagnosis confirmation. Bronchoscopy is commonly described as having a “rock garden” or “cobblestone” appearance [8]. A pulmonary function test also aids in the diagnosis. Differential diagnosis includes nodular sarcoidosis, amyloidosis, relapsing polychondritis, and respiratory papillomatosis.

In our case, the patient’s pulmonary function test, CT scan, flexible bronchoscopy, and histopathology helped with the accurate diagnosis of TO. Treatment options are very limited. In most cases, symptomatic management works well. Surgical resection of the affected trachea/bronchi is usually not feasible in extensive diseases like our patient but can be considered in the localized disease process. Other potential options include radiotherapy and bronchoscope interventions. Radiotherapy has shown marginal success. Bronchoscopic treatment modality is the least invasive option with effective treatment. Rigid bronchoscopy with neodymium-doped yttrium aluminum garnet (Nd: YAG) laser and stents can result in an effective and permanent cure of this potentially debilitating disease. First, laser treatment is performed on these calcified nodules, which is followed by mechanical debulking. Lastly, a silicone stent, which is the optimal stent for benign diseases such as TO, should be used if necessary.

Extensive involvement of the trachea in tracheobronchopathia osteochondroplastica makes it one of the most difficult airway obstructions to manage. A well-equipped interventional bronchoscopy facility and a well-trained interventional pulmonologist can offer the safest and long-term treatment management of this disease process [9].

## Conclusions

Tracheobronchopathia Osteochondroplastica (TO) is a nonmalignant condition that affects the central airways and spares the terminal/peripheral airways. Clinical presentation varies from person to person and ranges from being completely asymptomatic to wheezing, hemoptysis, recurrent infections, cough, and shortness of breath. A careful history and physical examination along with diagnostic studies which include pulmonary function test, CT scan of the chest, and bronchoscopy help in making an accurate diagnosis of this rare entity. Bronchoscopy appears to be key in diagnosis and excluding other conditions with similar presentations. The presence of nodular calcifications on CT scan should raise suspicion and confirmation can be done with flexible bronchoscopy. Treatment also varies as symptoms from person to person. Asymptomatic patients are closely monitored and patients with severe symptoms like ours should undergo interventions to improve their quality of life. For our patient, since she was symptomatic and had an extensive disease process, we decided to treat her. She underwent serial bronchoscopies with Nd:YAG laser treatment to the calcified nodules, which was followed by mechanical debulking with complete resolution of the abnormalities in the trachea.Consulting physician’s awareness about this disease process/condition is very important in its diagnosis and management.

## References

[1] Abu-Hijleh M, Lee D, Braman SS (2008). Tracheobronchopathia osteochondroplastica: a rare large airway disorder. Lung.

[2] Ulasli SS, Kupeli E (2015). Tracheobronchopathia osteochondroplastica: a review of the literature. Clin Respir J.

[3] Wilks S (1857). Ossific deposits on larynx, trachea and bronchi. Trans Pathol Society London.

[4] Jabbardarjani HR, Radpey B, Kharabian S, Masjedi MR (2008). Tracheobronchopathia osteochondroplastica: presentation of ten cases and review of the literature. Lung.

[5] Rana A, Mezughi H, Malik SA, Mansoor K, Al-Astal A (2020). Rare manifestation of idiopathic tracheobronchopathia osteochondroplastica: misdiagnosed and untreated entity?. Cureus.

[6] Virchow R (1863). Die krankhaften geschwülste. Berlin Hirschwald.

[7] Aschoff-Freiburg L (1910). Ueber Tracheopathia Osteoplastica. Verh Dtsch Gesch Pathol.

[8] Meyer CN, Dossing M, Broholm H (1997). Tracheobronchopathia osteochondroplastica. Respir Med.

[9] Dutau H, Musani A (2004). Treatment of severe tracheobronchopathia osteochondroplastica. J Bronchol Interv Pulomonol.

